# An innovative scoring system for predicting an excellent Harris hip score after proximal femoral nail anti-rotation in elderly patients with intertrochanteric fracture

**DOI:** 10.1038/s41598-022-24177-7

**Published:** 2022-11-19

**Authors:** Ong-art Phruetthiphat, Panukorn Pinijprapa, Yodhathai Satravaha, Nitchanant Kitcharanant, Chatlert Pongchaiyakul

**Affiliations:** 1 Department of Orthopaedics, Phramongkutklao Hospital and College of Medicine, 315 Ratchvidhi Rd, Thung Phaya Thai, Ratchathewi, Bangkok, 10400 Thailand; 2 Department of Orthodontics, Faculty of Dentistry, Mahidol University, Bangkok, Thailand; 3 Department of Orthopaedics, Chiang Mai University, Chiang Mai, Thailand; 4 Division of Endocrinology and Metabolism, Department of Medicine, Faculty of Medicine, Khon Kaen University, Khon Kaen, Thailand

**Keywords:** Health care, Medical research

## Abstract

Typically, intramedullary and extramedullary devices are used to treat elderly with intertrochanteric fractures. The majority of previous research has focused on the association between surgical factors and mechanical failure after internal fixation. There is, however, limited evidence to demonstrate the association between functional outcomes after proximal femoral nail anti-rotation (PFNA) fixation and the non-surgical factors such as patient’s comorbidities. The aim of this study is to determine the predictive factors associated with excellent outcome, as well as to develop an integrated scoring system to predict the outcome after PFNA fixation in elderly patients with an intertrochanteric fracture. A retrospective study was conducted between January 2012 and December 2018. Elderly patients with low-energy intertrochanteric fractures who underwent PFNA fixation and at least a year of follow-up were recruited. Demographics, comorbidities, cognitive status, time to operation, and surgical parameters of the patients were all identified. Excellent and non-excellent outcomes were assessed by Harris Hip Score (HHS) after a one-year follow up. Regression analysis was used to determine the predictors for an excellent functional outcome. A new integrated scoring system (ISSI; Integrate Scoring System in elderly patients with Intertrochanteric fracture) was developed and validated. 450 elderly patients were randomly divided into two cohorts: a development (N = 225) and validation cohorts (N = 225). In this study, age < 85 years, normal weight/overweight, Charlson comorbidity index (CCI) < 6, no cognitive impairment, a modified AO/OTA 31A1.3, time to operation < 6 days, and Tip Apex Distance between 20 and 30 mm were significantly associated with an excellent outcome after PFNA fixation. The range of ISSI score was between 0 to 16 and the cut-off score of 13 was found to have the highest discriminatory power to determine the excellent functional outcome where the area of ROC was 0.85. In regards to the validation cohort, the sensitivity and specificity of ISSI score was 69% and 87%, respectively, and the AUC was 0.81. The ISSI score is effortless and practical for orthopedic surgeons for predicting an outcome after PFNA fixation in elderly patients with an intertrochanteric fracture.

## Introduction

Intertrochanteric fractures are common fractures in patients with osteoporosis, and a major public health concern because of the considerably great risk of mortality, morbidity, functional impairment and incurs significant cost^1–3^. Frisch et al. demonstrated that the 90-days mortality rate for intertrochanteric fractures was 12.1%^4^ while a systematic review reported that the 1-year mortality rate after intertrochanteric fracture was 17.5%^5^. Furthermore, a recent study found that older age (more than 75 years old), higher comorbidity (Charlson Comorbidity Index (CCI) at least 3), and not receiving zoledronic acid were associated with increased mortality^6^. The surgical options for intertrochanteric fractures are generally composed of intramedullary and extramedullary devices^7–9^. Although, stable type intertrochanteric fractures can be treated with intramedullary or extramedullary devices (Dynamic hip screw; DHS); however, a higher rate of proximal femur shortening has been reported following treatment with DHS^8^. Furthermore, previous studies advocated fixation of AO/OTA classification type 31A2 and 31A3 for unstable type intertrochanteric fracture with intramedullary device^9^, and mainly focused on the association of surgical parameters and mechanical failure after internal fixation^10–14^. However, there is limited data which demonstrates the association between functional outcomes after proximal femoral nail anti-rotation (PFNA) fixation and the non-surgical factors including patient’s comorbidities. Moreover, individuals with intertrochanteric fractures should be mobilized as soon as possible and early ambulation be performed after operation to minimize the mortality^15^.

We hypothesize that not only surgical factors are responsible for the excellent outcomes after PFNA fixation, but also including non-surgical parameters, preinjury ambulatory status, cognitive impairment, and time to operation. Therefore, this present study is aimed to identify the predictive factors associated with an excellent outcome after PFNA fixation, and to develop and validate a new integrated scoring system to predict the outcome after PFNA fixation in elderly patients with an intertrochanteric fracture.

## Patients and methods

### Setting and subjects

After the ethics committee approved the study protocol (R116h/62_Exp), a retrospective study was conducted at the Department of Orthopaedics, Phramongkutklao Hospital which is a tertiary care trauma center, during a period of seven years from January 2012 to December 2018. Elderly patients aged over 60 years suffering from intertrochanteric fracture from low energy trauma who underwent PFNA fixation with at least a 1 year follow up period after operation were recruited. Patients with a pathological fracture on the basis of a primary tumor, metastasis, history of high energy trauma, multiple fractures, referred to other hospitals after operation, or incomplete medical records were excluded.

### Measurements

Demographic data were recorded and obtained from the electronic medical record, including age, gender, body weight and height, underlying diseases (including diabetes mellitus, hypertension, dyslipidemia, cardiovascular disease, chronic kidney disease, gout, cognitive impairment), current medications, surgical parameters, functional outcomes and complications. Body mass index (BMI) was calculated and divided into three groups based on body weight in kilograms and height in squared meters (kg/m^2^). Underweight is defined as having a BMI of less than 18.5 kg/m^2^, normal weight as having a BMI of 18.5 to less than 25 kg/m^2^, and overweight as having a BMD of 25 to less than 30 kg/m^2^. In addition, Charlson Comorbidity Index (CCI)^16,17^ and the American Society of Anesthesiologists (ASA) physical status classification^18^ were used to assess patients' physical condition before surgery as shown in Table 1. Cognitive impairment was evaluated by mini-mental state examination (MMSE)^19^. This tool composed of short/long term memory, attention span, concentration, language and communication skills, ability to plan, and ability to understand instructions. Those patients with MMSE less than 25 were classified as “Cognitive impairment” while those patients with MMSE between 25 and 30 were not cognitively impaired.

**Table 1:** Clinical characteristics and comorbidity of patients with intertrochanteric fracture.

Parameters	Total (N = 450)	Development group (N = 225)	Validation group (N = 225)	p-value
**Demography**				
Age (years)				0.843
< 85	294 (65.3%)	146 (64.9%)	148 (65.8%)	
≥ 85	156 (34.7%)	79 (35.1%)	77 (34.2%)	
Mean ± SD	80.6 ± 8.5	80.7 ± 8.6	80.6 ± 8.4	0.838^+^
Median (Min–Max)	82 (60–102)	82 (60–99)	82 (60–102)	
Gender				0.753
Male	127 (28.2%)	163 (72.4%)	160 (71.1%)	
Female	323 (71.8%)	62 (27.6%)	65 (28.9%)	
Weight (kg)	55.2 ± 11.1	54.7 ± 11.3	55.7 ± 10.8	0.329
Height (cm)	156.9 ± 8.5	156.9 ± 7.9	156.8 ± 8.1	0.886
BMI (kg/m^2^)				0.115
Underweight	70 (15.6%)	43 (19.1%)	27 (12.0%)	
Normal weight	288 (64.0%)	44 (19.6%)	48 (21.3%)	
Overweight	92 (20.4%)	138 (61.3%)	150 (66.7%)	
Mean ± SD	22.4 ± 3.8	22.2 ± 4.0	22.6 ± 3.6	0.247^+^
Median (Min–Max)	22.2 (13.3–41.9)	22.1 (14.0–41.9)	22.2 (13.3–33.3)	
**Comorbidity**				
ASA class				0.414*
1	9 (2.0%)	3 (1.3%)	6 (2.7%)	
2	131 (29.1%)	70 (31.1%)	61 (27.1%)	
3	310 (68.9%)	152 (67.6%)	158 (70.2%)	
CCI				
Mean ± SD	4.7 ± 1.6	4.5 ± 1.5	4.9 ± 1.7	**0.013**^**+**^
Median (Min–Max)	4 (2–10)	4 (2–9)	5 (2–10)	**0.034**^**++**^

*BMI* body mass index, *CCI* Charlson comorbidity index, *cm* centimeter.

Chi-square; *Fisher's exact test; ^+^Independent *t* test; ^++^Mann–Whitney *U* test. Significant values are in bold.

Surgical parameters including Tip Apex Distance (TAD) (millimeter, mm)^12^, surgical time (minute) and blood loss (milliliter, mL), time to operation (day), and fracture patterns were recorded. In this study, fracture patterns based on modified AO/OTA classification were retrospectively categorized into AO/OTA 31 A1.3, A2.2, and A2.3.

### Surgical procedure and postsurgical management

In the present study, all intertrochanteric fractures were fixed with a titanium PFNA™ nail (Synthes). All patients were operated on the fracture table in the supine position. Closed reduction was performed under fluoroscopy. After anatomical reduction, a guide wire was inserted into the tip of greater trochanter, proximal reaming was done, diameter of nail was measured under fluoroscopy, and a standard-proximal femoral nail anti-rotation (PFNA™) with 200 mm length was placed into the medullary canal, then the guide wire was removed. Before applying the helical blade into the femoral head, a guide wire was inserted into the femoral head with the exact position, and lengths of helical blade in AP and lateral views were measured. The helical blade was inserted into the femoral head and it was tightened in the next step, then a distal screw was finally applied. After the operation, an appropriate pain control was provided for all patients. When patients had no or mild pain (visual analogue score < 4) on the affected hip, they were allowed to bear weight as tolerated, and deep vein thrombosis prophylaxis (with mechanical pump or anticoagulant) was prescribed in all patients.

### Outcome measurements

All patients were followed in clinic at 2 weeks, 4 weeks, 6 weeks, 3 months, 6 months, 9 months, and 1 year after discharge from the hospital. Plain radiographic examinations of both hips (anteroposterior (AP) radiographs of both hips and lateral radiographs of affected hip) were performed at a 2-week follow up and measured by two independent orthopedic training surgeons who did not participate in the surgical procedures. PACS software was used to assess the quality of reduction including neck-shaft angle (NSA), displacement between cortices of proximal and distal fragments, gap, step and Tip Apex Distance (TAD) in AP and lateral views (in millimeter, mm)^12^. The average of the measurements taken by two orthopedic surgeons was then computed.

The functional outcome was assessed using Harris Hip Score (HHS) which was divided into two aspects in all patients: pre-fracture state via interview and postsurgical state via clinic examination at one year follow-up. In summary, HHS is made up of many components such as pain (44 points), limp (11 points), support (11 points), distance walked (11 points), sitting (5 points), entering public transportation (1 point), stairs (4 points), putting on socks and shoes (4 points), absence of deformity (4 points), and range of motion (5 points). Zero point represents the lowest HHS while one hundred points represents the highest HHS^20^. In this study, HHS were divided into two categories; excellent (90–100 points) and non-excellent outcomes (< 90 points). Furthermore, any patients who died within the first year of follow-up were classified into non-excellent outcome. The fracture pattern, TAD, surgical factors, and functional outcome as determined by HHS were shown in Table 2. In addition, the percentage of excellent and non-excellent outcomes by surgical factors (fracture pattern and TAD) were shown in Table 3.

**Table 2:** Fracture pattern, surgical factors, and functional outcome (HHS at 1 year).

Parameters	Total (N = 450)	Development group (N = 225)	Validation group (N = 225)	p-value
**Fracture pattern** (**modified AO/OTA)**				
Type 31A1.3	194 (43.1%)	97 (43.1%)	97 (43.1%)	1.000
Type 31A2.2	180 (40.0%)	90 (40.0%)	90 (40.0%)	
Type 31A2.3	76 (16.9%)	38 (16.9%)	38 (16.9%)	
**Tip apex distance (TAD) (mm)**				
> 30	20 (4.4%)	13 (5.78%)	7 (3.1%)	0.302
< 20	131 (29.1%)	68 (30.22%)	63 (28%)	
20–30	299 (66.5%)	144 (64%)	155 (68.9%)	
**Surgical factors**				
Surgical time (min)				
Mean ± SD	69.6 ± 19.7	69.0 ± 18.7	70.1 ± 20.7	0.567^+^
Median (Min–Max)	65 (40–180)	65 (40–150)	65 (40–180)	
Estimated blood loss (ml)				
Mean ± SD	99.7 ± 93.4	101 ± 86.9	98.4 ± 99.6	
Median (Min–Max)	50 (20–700)	100 (20–550)	50 (20–700)	0.132^++^
**Harris Hip Score (HHS)**				0.345
Excellent (≥ 90)	234 (52.0%)	122 (54.2%)	112 (49.8%)	
Non-excellent (< 90) + mortality 1 year	216 (48.0%)	103 (45.8%)	113 (50.2%)	
Mortality 1 year	4 (1.8%)	3 (2.9%)	1 (0.9%)	0.447
Mean ± SD	87.1 ± 7.9	87.7 ± 7.5	86.6 ± 8.2	0.150^+^
Median (Min–Max)	90 (54–99)	90 (57–99)	90 (54–98)	

Chi-square test; ^+^Independent *t* test; ^++^Mann–Whitney *U* test.

**Table 3:** Percentage of excellent and non-excellent outcomes by surgical factors.

Operation factors	Excellent outcome (N = 234)	Non-excellent outcome (N = 216)	p-value
**Modified AO/OTA**			**< 0.001**
Type 31A1.3*	129 (55.1%)	65 (30.1%)	
Type 31A2.2	84 (35.9%)	96 (44.4%)	**< 0.001**
Type 31A2.3	21 (9.0%)	55 (25.5%)	**< 0.001**
**TAD (mm)**			**< 0.001**
20–30*	156 (66.7%)	143 (66.2%)	
> 30	2 (0.8%)	18 (8.3%)	**< 0.001**
< 20	76 (32.5%)	55 (25.5%)	0.263

*Reference; *TAD* Tip Apex Distance, *mm* millimeters.

The outcome assessed by HHS. Significant values are in bold.

### Statistical analysis

STATA version 14.0 was used for all statistical analyses (StataCorp., College Station, TX, USA). Descriptive statistics are used to categorize and summarize demographic data. Categorical data were presented as percentages or proportions. Continuous data were presented as means with standard deviations (SD) or medians with minimum and maximum value, as appropriate. Comparisons of categorical variables were made using the Chi-squared or Fisher’s exact test, as appropriate. Continuous variables were tested for the normality using a Shapiro–Wilk test and were compared using the Student’s *t* test or Mann–Whitney *U* test, as appropriate.

### Development and validation the integrate scoring system

**Figure 1:**
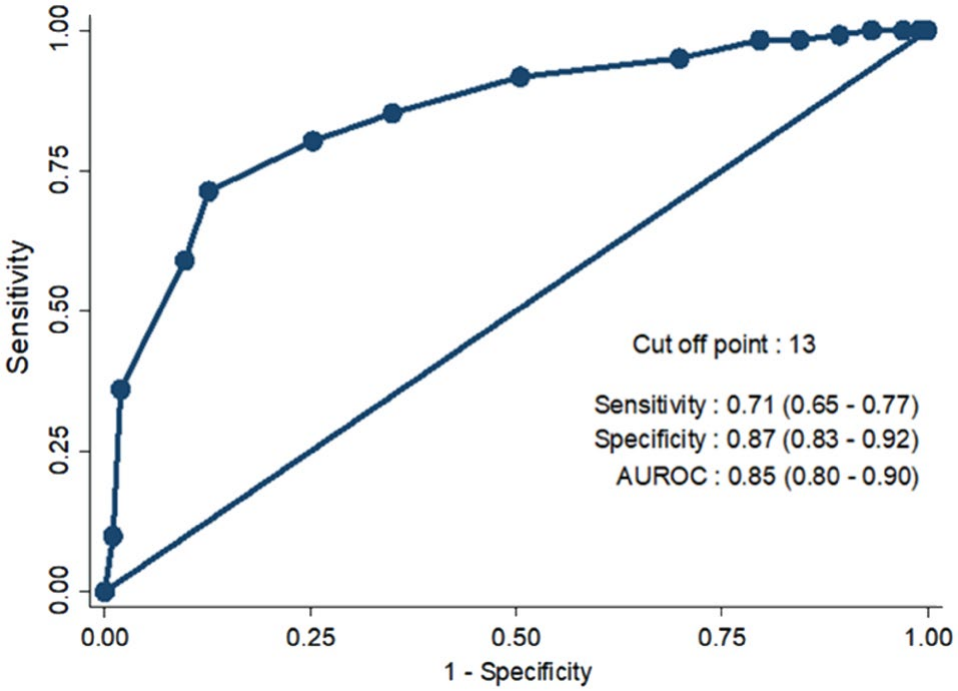
The ROC curve of ISSI score in predicting an excellent outcome in development cohort.

A new integrate scoring system (ISSI; Integrate Scoring System in elderly patients with an Intertrochanteric fracture) was developed and validated. The entire sample were randomly divided into two datasets in a 1:1 ratio; development cohort and validation cohort. The odds ratio (OR) with 95% confidence interval (95% CI) and p-value were used to determine the association between predictors and excellent outcome. The variables with a p-value < 0.20 based on the results of univariate analysis and clinically significant variables were entered into a multivariate logistic regression model. Backward selection was performed to choose a subset of the predictor variables for the final model as demonstrated in Table 4. The score-based predictive model was created from the logistic regression equation using the regression coefficient-based scoring method to predict an excellent outcome. To generate a simple integer-based point score for each predictor, the scores were calculated by dividing beta coefficients by the absolute value of the smallest coefficient in the model and rounding up to the nearest integer. The total score of ISSI for each patient was calculated by adding each component together, then the receiver operating characteristic (ROC) curve analysis and the area under the ROC curves (AUC) were computed. A cut-off ISSI score with the highest discriminatory power was derived from the ROC curves, and the highest Youden’s index was used in the interpretation and evaluation of a score with maximum effectiveness (Fig. 1). In validation cohort, a cut-off score was tested. The scoring system (ISSI) was used to determine each patient’s score. The sensitivity and specificity were all calculated.

**Table 4:** Univariate and multivariate analysis, and predictive score for excellent outcome in development cohort.

Factors	Excellent (HHS ≥ 90) N (%)	Non-excellent (HHS < 90) N (%)	Crude OR	Adjusted OR* (95% CI)	p-value	Coefficient	Predictive score
**Age**							
< 85 years	92 (75.4%)	54 (52.4%)	2.78	2.43 (1.18–5.00)	**0.016**	0.89	**2**
≥ 85 years	30 (24.6%)	49 (47.6%)	1.00	1.00			0
**BMI**							
Normal or overweight	109 (89.3%)	73 (70.9%)	3.45	7.02 (2.97–16.60)	**< 0.001**	1.95	**4**
Underweight	13 (10.7%)	30 (29.1%)	1.00	1.00			0
**CCI**							
< 6	113 (92.6%)	59 (57.3%)	9.36	9.74 (4.00–23.71)	**< 0.001**	2.28	**4**
≥ 6	9 (7.4%)	44 (42.7%)	1.00	1.00			0
**Cognitive impairment**							
No	113 (92.6%)	71 (68.9%)	5.66	3.43 (1.29–9.08)	**0.013**	1.23	**2**
Yes	9 (7.4%)	32 (31.1%)	1.00	1.00			0
**Time to operation**							
< 6 days	54 (44.3%)	34 (33.0%)	1.61	2.24 (1.09–4.59)	**0.027**	0.80	1
≥ 6 days	68 (55.7%)	69 (67.0%)	1.00	1.00			0
**Modified AO/OTA classification**							
Type 31A1.3	69 (56.6%)	28 (27.2%)	6.05	3.09 (1.13–8.48)	**0.028**	1.13	**2**
Type 31A2.2	42 (34.4%)	48 (46.6%)	2.15	1.73 (0.64–4.70)	0.282	0.55	**1**
Type 31A2.3	11 (9.0%)	27 (26.2%)	1.00	1.00			0
**TAD**							
20–30	84 (68.8%)	60 (58.3%)	1.58	1.71 (0.86–3.43)	0.128	0.54	**1**
< 20 or > 30	38 (31.2%)	43 (41.7%)	1.00	1.00			0

*OR* Odds Ratio, *95% CI* 95% confidential interval, *HHS* Harris Hip Score, *1.00* Reference.

*Adjusted for Age, BMI, Cognitive impairment, DM, Hypertension, Dyslipidemia, Chronic kidney disease, CCI, vitamin D, Operative time, Operative blood loss, TAD, Modified AO/OTA, Length of hospital stay. Significant values are in bold.

### Ethical approval and consent to participate

The study was approved by the Institutional Review Board Royal Thai Army Medical Department (IRBRTA). All procedures were performed in accordance with relevant guidelines. This study was registered to IRBRTA and it has been approved with a code of R116h/62_Exp.

### Informed consent

Informed consent was obtained from all individual participants included in the study.

## Results

A total of 729 intertrochanteric fractures from low energy trauma in patients over the age of 60 were reviewed retrospectively. This study excluded 28 patients with primary tumor diagnoses, 15 patients with metastasis, 56 patients from high energy trauma, 9 patients with multiple fractures, 42 patients referred to another hospital after surgery, and 129 patients with incomplete medical record. Finally, this study enrolled 450 patients. Mean age and BMI were 80.6 ± 8.5 years (range, 60–102) and 22.4 ± 3.8 kg/m^2^ (13.3–41.9), respectively. There was no significant difference in age, gender, weight, height body mass index, and ASA class between the development and validation group; however, CCI was significantly higher in validation group.

Fracture pattern assessed by modified AO/OTA was 43.1%, 40.0% and 16.9% from 31A1.3, 31A2.2, and 31A2.3, respectively. In this study, around two-thirds of patients (299/450, 66.5%) had TAD in acceptable range (20–30 mm), whereas one-third of patients (33.5%) had TAD in unacceptable range (> 30 or < 20 mm). According to HHS, 234 (52%) and 216 (48%) were classified in excellent and non-excellent outcome group, respectively. There was no significant difference in fracture pattern, TAD, operation times, blood loss, functional outcome (HHS), and one-year mortality between development and validation groups (Table 2). We found that patients with excellent outcome had a significant higher prevalence of type 31A1.3, but lower prevalence of type 31A2.2/2.3 than those with non-excellent outcome. However, there was no difference in TAD in acceptable range between excellent and non-excellent outcome group (Table 3).

### Development and validation of ISSI score

In unadjusted analysis, younger age (< 85 years), normal or overweight, CCI < 6, no cognitive impairment, modified AO/OTA type 31A1.3, time to operation < 6 days, and TAD in acceptable range (20–30 mm) were significantly associated with an excellent outcome. The association remained unchanged (except TAD) after multivariate adjustment. The range of ISSI score was between 0 to 16 and the cut-off score of 13 was found to have the highest discriminatory power to determine the excellent functional outcome. Subsequently, for identifying excellent outcome after PFNA fixation in elderly women with intertrochanteric fracture, the ISSI score (≥ 13) had the sensitivity and specificity of 71% and 87%, respectively. The ISSI score system yield an AUC of 0.85 (95% CI 0.80–0.90) (Fig. 1). When applied ISSI score to the validation cohort, the sensitivity was 69% and specificity was 87%, and the AUC was 0.81 (95% CI 0.76–0.87).

## Discussion

To prevent mortality in elderly, standard treatment after surgical fixation for hip fracture should be followed by mobilization as soon as possible^15^. However, there was limited evidence linking functional outcomes following PFNA fixation to non-surgical factors including patient’s comorbidities. This study found that both clinical indicators and surgical characteristics, patients who age < 85 years, normal or overweight, CCI < 6, no cognitive impairment, had a modified AO/OTA type 31A1.3, time to operation < 6 days, and had a TAD in the acceptable range (20–30 mm) were significantly associated with an excellent outcome following PFNA fixation in elderly patients with an intertrochanteric fracture; as well as construct and verify a new prediction score for excellent outcome in development and validation cohorts.

Many non-surgical factors were associated with the functional outcomes after PFNA fixation, according to our findings. The most important predictors of an excellent outcome after PFNA fixation were age, BMI, CCI, cognitive status, and time to operation. Our findings found that a younger age (less than 85 years), a normal BMI or overweight, a lower CCI (less than 6), no cognitive impairment, and early surgery were strongly associated with a positive surgical outcome, which were consistent with earlier studies^21–26^. Previous studies found patients’ pre-injury function^21^ and cognitive status^22^ of the patients prior to surgery, were determined by their age. Low BMI was associated with more blood transfusions^23^, which was related to a longer hospital stay and an increased risk of hematogenous infection^24^, whereas patients with fewer comorbidities had shorter hospital stays^25^ and were more likely to mobilize sooner^27^. Furthermore, patients without cognitive impairment have a better chance of mobilizing faster and returning to their pre-injury status sooner^26^, as well as having a lower risk of developing postsurgical complications^22^.

In term of surgical parameters, previous studies reported that a quality of fracture reduction, position of blade, and TAD were associated with the outcome following PFNA fixation^12–14,28^. Kaufer et al. found that both uncontrollable and controllable factors affect mechanics after intertrochanteric fracture surgery^28^. Baumgaertner et al. demonstrated the correlation between TAD and mechanical failure^12,13^, while Bojan et al. found that screw cut-out after Gamma nail treatment for proximal femoral fractures was associated with unstable fracture, non-anatomical reduction, and non-optimal screw position^14^. The current study confirmed prior findings that patients with a poor outcome had a considerably larger TAD of more than 30 mm (p < 0.001) and a significantly higher rate of unstable type intertrochanteric fracture (p < 0.001). Furthermore, we found that having a modified AO/OTA 31A1.3 and an appropriate TAD were linked to a higher rate of excellent functional outcome following PFNA fixation. In addition, stable type (a modified AO/OTA 31A1.3) was found to be related with outstanding results.

For intramedullary nailing intertrochanteric fractures, anatomical reduction, suitable TAD and lag screw position, and stable fracture type resulted in a higher stability score^12,13,27^. Hsu et al. developed an integrated scoring system for predicting the outcome of elderly intertrochanteric fractures after dynamic hip screws, and found that the AO/OTA 31-A2 classification, postsurgical lateral wall fracture, posteriorly inserted lag screw and varus reduction pattern were all significant risk predictors for DHS failure^29^, while Lee et al. demonstrated the stability score to predict the mechanical failure after intramedullary nailing fixation in elderly with an intertrochanteric fracture, and they found that combination of anatomical reduction, fixation skill, and Kyle’s classification type I–II significantly reduced the rate of fixation failure^27^. Most studies, however, did not look into medical conditions including CCI, cognitive impairment, and early ambulation. To our knowledge, this is the first study to include both surgical and non-surgical factors to develop an integrating scoring model (ISSI) for predicting an excellent outcome after PFNA fixation in the patients with intertrochanteric fracture. Combining the two characteristics could offer clinician with a comprehensive picture of the patient’s condition and underline the need of treating patients holistically. According to the current study, patients with an ISSI score ≥ 13 had a higher chance of having an excellent outcome after PFNA fixation.

This study should be interpreted in the context of a number of potential strengths and weaknesses. The strength of this study was the appropriate sample population and follow up time, which was divided into development and validation groups by randomized process and included both surgical and medical parameters in order to develop an integrated scoring system for predicting a 1-year outcome after surgical fixation. There were, however, a few limitations as following; (1) this integrating scoring system was developed by retrospective data which could have biased the results to some extent; (2) we did not include bone mineral density (BMD) results in the scoring system because half of the patients did not have this test performed. Third, despite the fact that the study patients were randomly selected, well-characterized, and a large sample size; however, the study patients were Thai, among whom body frame, lifestyles and environmental factors are different from other population. Furthermore, a postoperative pain control, rehabilitation program and medical care system in this study differ from other settings. As a result, external validation is required before a model can be extrapolated to other populations).

## Conclusions

This integrated scoring system (ISSI score) was developed from surgical and medical characteristics for predicting a good result in elderly patients with an intertrochanteric fracture treated with PFNA fixation. Orthopedic surgeons can utilize the score to evaluate and treat patients since it is sensitive and specific.

## Data Availability

Requests for data not shown in the body of this manuscript can be made to the corresponding author.

## Abbreviations

PFNA: Proximal femoral nail anti-rotation
HHS: Harris hip score
ISSI: Integrated scoring system in elderly patients with Intertrochanteric fracture
DHS: Dynamic hip screw
BMI: Body mass index
CCI: Charlson comorbidity index
ASA: The American Society of Anesthesiologists physical status classification
MMSE: Mini-mental state examination
TAD: Tip apex distance
AP: Anteroposterior
NSA: Nail shaft angle
mm: Millimeter
OR: Odd ratio
ROC: Receiver operating characteristic
AUC: Area under the curve
